# Case Report: Monotypic IgG1-λ fibrillary glomerulonephritis associated with rheumatoid arthritis

**DOI:** 10.3389/fneph.2026.1932311

**Published:** 2026-10-13

**Authors:** Adam Nedoroscik, Jana Moravcova, Lenka Manakova, Oskar Zakiyanov, Romana Rysava, Vladimir Tesar, Marek Kollar

**Affiliations:** 1 Clinical and Transplant Pathology Department, Institute for Clinical and Experimental Medicine, Prague, Czechia; 2 Institute of Pathology, First Faculty of Medicine, Charles University and General University Hospital in Prague, Prague, Czechia; 3 Department of Pathology, AGELLAB, Novy Jicin, Czechia; 4 Institute of Physiology, First Faculty of Medicine, Charles University, Prague, Czechia; 5 Department of Nephrology, First Faculty of Medicine, Charles University and General University Hospital in Prague, Prague, Czechia

**Keywords:** case report, FGN, fibrillary glomerulonephritis, monotypic GN, organized deposit

## Abstract

**Background:**

Fibrillary glomerulonephritis (FGN) is a rare glomerular disease characterized by non-amyloid fibrillary deposits and is identified in fewer than 1% of native kidney biopsies. Most cases demonstrate polytypic immunoglobulin G deposition, whereas confirmed monotypic FGN is exceptionally uncommon and may raise concern for an underlying monoclonal gammopathy.

**Case presentation:**

A 72-year-old woman with rheumatoid arthritis presented with nephrotic-range proteinuria (4.6 g/24 h), microscopic hematuria, hypoalbuminemia, lower-extremity edema, and mildly impaired kidney function. Kidney biopsy demonstrated mesangial expansion and glomerular deposits staining for IgG, C3, and lambda light chain. The deposits were Congo red negative and showed strong, diffuse glomerular DNAJB9 positivity. Electron microscopy revealed randomly oriented, non-branching fibrils measuring approximately 20 nm, accompanied by podocyte foot-process effacement. Isolated IgG1 staining and lambda restriction, confirmed by immunofluorescence performed on pronase-digested paraffin-embedded tissue, supported a true monotypic IgG1-lambda phenotype. Serum immunofixation and serum free light-chain testing did not demonstrate a monoclonal protein, and no underlying hematological malignancy, other malignancy, or infection was identified. The patient received rituximab. At 14 months, proteinuria had decreased to approximately 1.0 g/24 h and serum albumin had increased from 30 to 38 g/L.

**Conclusion:**

Confirmed monotypic FGN is exceptionally rare and requires integration of routine immunofluorescence, IgG-subclass assessment, paraffin-embedded immunofluorescence, DNAJB9 immunohistochemistry, and electron microscopy. Light-chain restriction alone is insufficient to establish a truly monotypic process, and monotypic deposits warrant careful evaluation for an underlying clonal disorder. This case documents DNAJB9-positive monotypic IgG1-lambda FGN occurring in the setting of rheumatoid arthritis without demonstrable monoclonal gammopathy.

## Introduction

The kidneys maintain systemic homeostasis by removing metabolic waste products while retaining essential plasma proteins. Selective ultrafiltration occurs across the glomerular filtration barrier (GFB), which consists of three interconnected components: fenestrated glomerular endothelial cells, the glomerular basement membrane (GBM), and podocytes (1, 2). Podocytes are highly specialized visceral epithelial cells with interdigitating foot processes connected by slit diaphragms. The slit diaphragm is a specialized cell–cell junction composed of interacting structural and signaling proteins, including nephrin, Neph1, podocin, and CD2-associated protein and represents an essential component of the glomerular perm-selectivity barrier (1–3). Injury affecting the glomerular endothelium, GBM, podocyte foot processes, or slit diaphragm may compromise filtration-barrier integrity, permit the passage of plasma proteins into the urinary space, and consequently result in proteinuria.

Fibrillary glomerulonephritis (FGN) is a rare glomerular disease characterized by the deposition of non-amyloid fibrils, predominantly within the glomeruli (4). It was first described by Rosenmann and Eliakim in 1977 and accounts for approximately 0.5%–1% of native kidney biopsies (5). Clinically, FGN commonly presents with proteinuria, frequently in the nephrotic range, microscopic hematuria, hypertension, and impaired kidney function (6). The disease may be associated with malignancies, monoclonal gammopathy, autoimmune disorders, or infections.

The diagnosis of FGN requires the integration of light microscopy (LM), immunofluorescence (IF), electron microscopy (EM), and, increasingly, immunohistochemistry (IHC) (7). Pathologically, FGN usually demonstrates mesangial and/or capillary-wall deposits that are Congo red negative and stain for polytypic IgG, frequently with C3 and both light chains (8–10). Ultrastructurally, FGN is characterized by randomly oriented, non-branching fibrils with an average diameter of approximately 18–20 nm (4). Immunotactoid glomerulopathy represents an important differential diagnosis but is typically characterized by larger microtubular deposits with hollow centers and a more organized arrangement. DNAJB9 immunohistochemistry is a highly sensitive and specific tissue biomarker for FGN, although rare DNAJB9-negative cases have been reported (11, 12).

Most cases of FGN demonstrate polytypic IgG deposition, whereas apparently light-chain-restricted or monotypic staining is uncommon. Light-chain restriction on routine IF alone does not necessarily establish a true monotypic process. Suspected monotypic FGN should therefore be further evaluated using IgG-subclass staining and IF on pronase-digested paraffin-embedded tissue to exclude masked polytypic deposits. A convincing monotypic phenotype is supported by concordant restriction to a single IgG subclass and a single light chain. This distinction is clinically important because monotypic deposits raise the possibility of an underlying monoclonal gammopathy and require differentiation from other monoclonal immunoglobulin-associated glomerular diseases (11).

Here, we present a rare case of monotypic IgG1-lambda FGN occurring in a patient with rheumatoid arthritis. The diagnosis was supported by isolated IgG1 staining, lambda restriction demonstrated by routine IF and confirmed by IF on pronase-digested paraffin-embedded tissue, diffuse glomerular DNAJB9 positivity, negative Congo red staining, and characteristic randomly oriented fibrils on EM. To our knowledge, this is the first reported case of DNAJB9-positive, confirmed monotypic IgG1-lambda FGN occurring in the setting of rheumatoid arthritis without demonstrable monoclonal gammopathy. The patient also experienced a clinically meaningful reduction in proteinuria temporally associated with rituximab treatment.

## Case presentation

### Clinical presentation

A 72-year-old woman was admitted to the Department of Nephrology, First Faculty of Medicine, Charles University and General University Hospital in Prague, because of nephrotic-range proteinuria, bilateral lower-extremity edema, microscopic hematuria, and an unintentional weight loss of 15 kg during the preceding 2 years. The precise duration of the edema, proteinuria, and microscopic hematuria before hospitalization was not documented. Her medical history included well-controlled hypertension, hypothyroidism treated with replacement therapy, and rheumatoid arthritis diagnosed approximately 5 years before the current admission. She had received methotrexate for 3 years; whether methotrexate was ongoing at the time of admission was not documented.

The patient had no family history of kidney disease and not known history of malignancy. Hepatitis B and C infections were excluded serologically. Her blood pressure was 130/80 mmHg, and pulmonary and cardiovascular examinations were unremarkable. Bilateral lower-extremity edema was graded as 3 +. Kidney ultrasonography showed normal-sized kidneys with preserved renal parenchyma and scattered cysts in the right kidney.

### Laboratory investigation

Laboratory examination demonstrated mildly impaired kidney function, with a serum creatinine concentration of 108 µmol/L. 24-h urine collection showed nephrotic-range proteinuria of 4.6 g/24 h, and serum albumin was decreased to 30 g/L. Urinary sediment demonstrated microscopic hematuria with dysmorphic erythrocytes. Perinuclear antineutrophil cytoplasmic antibodies and anti-phospholipase A2 receptor antibodies were tested twice and showed a negative result. Serum-free light-chain concentrations were within the reference ranges, and serum protein immunofixation showed no detectable monoclonal protein. Because no circulating monoclonal protein or other clinical evidence of a hematological malignancy was identified, bone marrow biopsy was not performed. Hemoglobin was mildly decreased at 106 g/L. Kidney biopsy was performed on the fifth day of hospitalization.

### Light microscopy

One renal cortical core containing 31 glomeruli was examined, of which 11 were globally sclerotic. The viable glomeruli showed focal segmental mesangial expansion and increased mesangial cellularity (**Figures 1A, B**). Two glomeruli contained fibrocellular crescents, without fibrinoid necrosis. Segmental glomerulosclerosis was present in five glomeruli. Interstitial fibrosis and tubular atrophy involved approximately 30%–35% of the renal cortex and were accompanied by a corresponding chronic inflammatory infiltrate. Arteries showed intimal fibrosis, and arterioles contained hyaline deposits. Congo red and Saturn red stains were negative.

**Figure 1:**
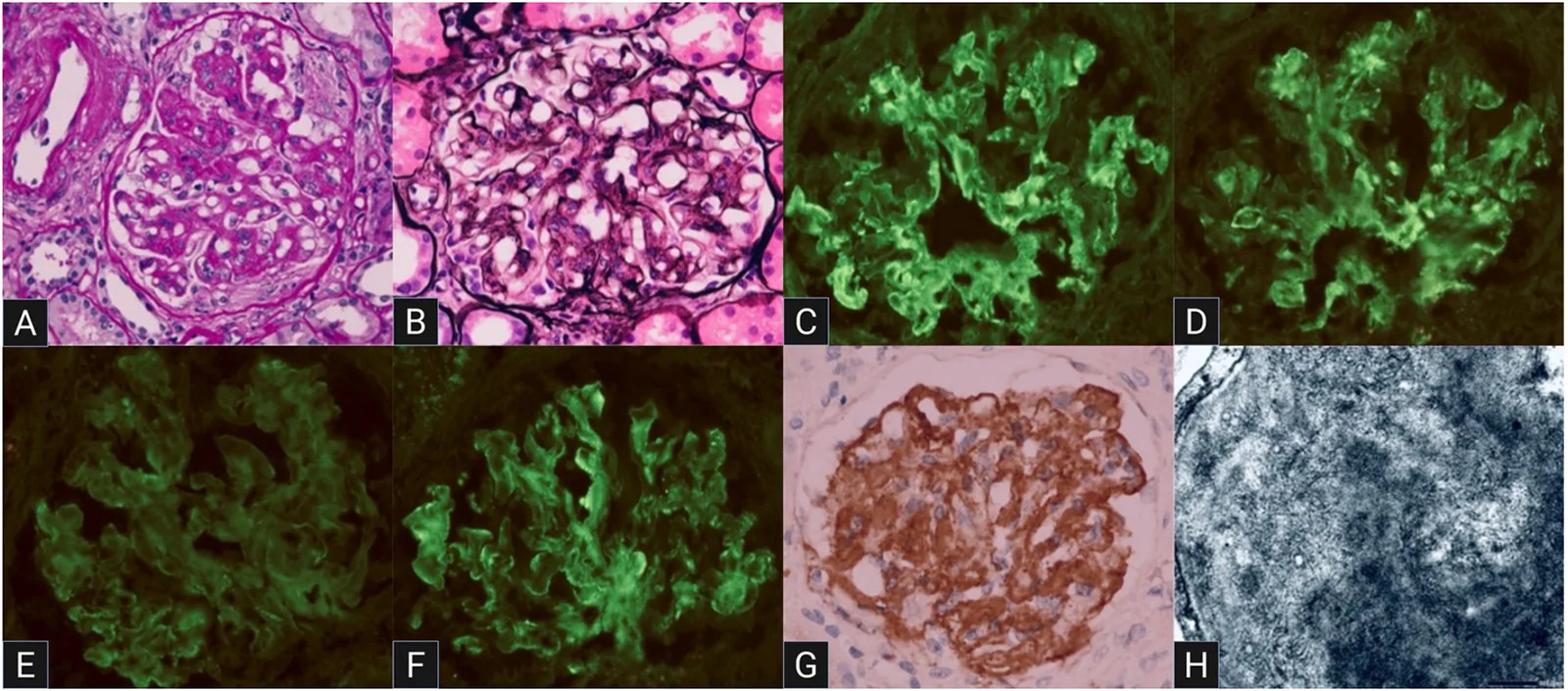
Histopathological, immunopathological, and ultrastructural findings in monotypic FGN **(A)** Periodic acid Schiff staining (LM, magnification 400×)—slightly enlarged glomerulus, expanded mesangium with segmentally increased mesangial cellularity and forming a fibrocellular crescent. **(B)** Silver methenamine staining (LM, magnification 400×)—slightly enlarged glomerulus. **(C)** IgG—positive (+++) global positivity in mesangium (magnification 400×). **(D)** IgG1—positive (+++) the same pattern as IgG (magnification 400×). **(E)** Kappa—negative (supporting lambda light-chain restriction) (magnification 400×). **(F)** Lambda—positive (++) the same pattern as IgG (magnification 400×). **(G)** DNAJB9 (immunohistochemistry, magnification 400×)—+++ strong positivity in mesangium and along the glomerular basement membrane. **(H)** Electron microscopy—randomly oriented, non-branching fibrils.

### Immunofluorescence microscopy

Routine IF demonstrated diffuse global granular staining for IgG (+++) and C3 (+++) in the mesangium and along the glomerular capillary walls. The lambda light chain showed moderate staining (++), whereas the kappa light chain exhibited a negative stain, suggesting an apparent lambda-restricted process. C1q showed mild staining (+),whereas IgA, IgM, and serum amyloid A stains were negative (**Figures 1C–F**).

### DNAJB9 immunohistochemistry

DNAJB9 immunohistochemistry showed strong, diffuse global staining of the mesangial and glomerular capillary-wall deposits, supporting the diagnosis of FGN (**Figure 1G**). No corresponding staining was identified in the tubulointerstitial compartment.

### Electron microscopy

EM demonstrated randomly oriented, non-branching fibrils measuring approximately 20 nm, together with podocyte foot-process effacement and segmental glomerulosclerosis (**Figure 1H**).

### Confirmation of the monotypic phenotype

Because routine IF suggested lambda light-chain restriction, additional IgG-subclass staining and IF on pronase-digested paraffin-embedded tissue were performed. The deposits showed isolated IgG1 positivity, whereas IgG2, IgG3, and IgG4 stains were negative. IF on pronase-digested paraffin-embedded tissue confirmed strong staining for IgG and lambda with persistent absence of kappa. The concordant restriction to IgG1 and lambda supported a true monotypic IgG1-lambda phenotype rather than apparent light-chain restriction caused by masked immunoglobulin staining.

Systemic evaluation showed no detectable monoclonal protein, hematological malignancy, other malignancy, or hepatitis B or C infection. Bone marrow biopsy was not performed because serum immunofixation and free light-chain testing provided no evidence of monoclonal gammopathy. The integrated findings supported DNAJB9-positive monotypic IgG1-lambda FGN without demonstrable monoclonal gammopathy.

### Treatment and follow-up

The case was discussed at a clinicopathological seminar, and therapy with rituximab was indicated. Rituximab was selected based on the limited published experience suggesting potential benefit from B-cell-directed therapy in some patients with FGN, particularly when an immune-mediated process is suspected (11, 13, 14). However, no standardized or definitively effective treatment for FGN has been established. The patient received an initial intravenous dose of 1 g at baseline, followed by a second 1-g dose 2 weeks later. A third dose was administered 6 months after treatment initiation. At diagnosis, serum creatinine was 108 µmol/L, 24-h urinary protein excretion was 4.6 g/24h, and serum albumin was 30 g/L. At the 14-month follow-up, proteinuria had decreased to approximately 1.0 g/24 h and serum albumin had increased to 38 g/L. Serum creatinine was 133 µmol/L, indicating persistent kidney dysfunction without progression to kidney failure during the observation period. The patient subsequently developed COVID-19 and died, with COVID-19 documented as the direct cause of death. An integrated graphical summary of the clinical presentation, diagnostic evaluation, final diagnosis, treatment, and outcome is provided in **Figure 2**.

**Figure 2:**
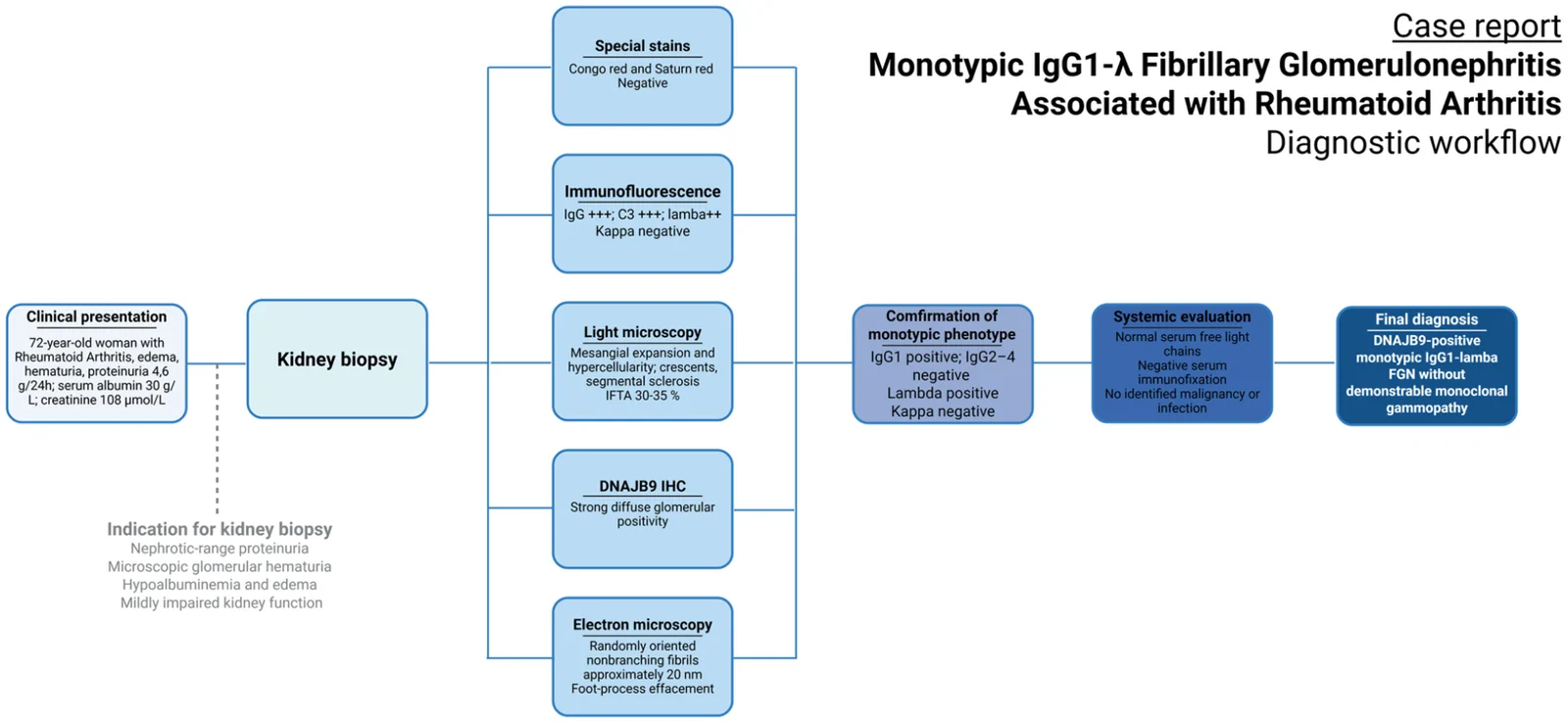
Diagnostic workflow for monotypic IgG1-λ fibrillary glomerulonephritis C3, complement component 3; DNAJB9, DnaJ heat shock protein family (Hsp40) member B9; FGN, fibrillary glomerulonephritis; IFTA, interstitial fibrosis and tubular atrophy; IgG, immunoglobulin G.

## Discussion

This case illustrates the clinicopathological presentation and diagnostic challenges of monotypic FGN, including its distinction from other organized or monoclonal deposits, evaluation for an underlying clonal process, association with rheumatoid arthritis, and clinical course following rituximab treatment.

### Principal findings

Glomerular IgG1-lambda-containing fibrils, mesangial expansion, and capillary-wall involvement were accompanied by podocyte foot-process effacement, indicating disruption of the glomerular filtration barrier. These findings explain the nephrotic-range proteinuria, hypoalbuminemia, and edema, whereas glomerular capillary-wall injury accounts for the dysmorphic hematuria. Mild kidney impairment likely reflected both active glomerular disease and chronic changes, including glomerulosclerosis, interstitial fibrosis and tubular atrophy, and vascular injury (1–4, 13, 15).

### Rationale for the diagnosis of monotypic FGN

Monotypic FGN should be distinguished from FGN showing apparent light-chain restriction on routine IF. Light-chain restriction demonstrated solely on frozen tissue does not necessarily establish a truly monotypic process because unequal staining sensitivity or masking of immunoglobulin epitopes may produce a falsely restricted pattern. Therefore, suspected monotypic FGN should be further evaluated using IgG-subclass staining and IF on pronase-digested paraffin-embedded tissue. A convincing monotypic phenotype is supported by concordant restriction to a single IgG subclass and a single light chain after masked polytypic deposits have been excluded (9, 16, 17). Importantly, the term “monotypic” describes the immunopathological composition of the glomerular deposits and does not, by itself, demonstrate an underlying clonal B-cell or plasma-cell disorder (16, 17).

The present case satisfied these pathological criteria. The deposits showed restriction to IgG1 and lambda, whereas the remaining IgG subclasses and kappa light chain showed negative results. Lambda restriction was demonstrated by both routine IF and IF performed on pronase-digested paraffin-embedded tissue, thereby reducing the possibility of an apparently restricted pattern caused by masked immunoglobulin staining. Furthermore, serum-free light-chain levels were normal, serum immunofixation showed a negative result, and no underlying hematological malignancy was identified. The findings therefore support true monotypic IgG1-lambda FGN without demonstrable monoclonal gammopathy.

Because the renal deposits demonstrated concordant IgG1 and lambda restriction, an underlying monoclonal gammopathy and the possibility of MGRS were considered. Serum protein immunofixation showed a negative result, and serum-free light-chain concentrations and the kappa-to-lambda ratio were within the reference ranges. The patient had no clinical or radiological evidence of a lymphoproliferative or plasma-cell malignancy. As no circulating monoclonal protein or other evidence of a hematological neoplasm was identified, bone marrow examination was not performed. These findings reduced the likelihood of a clinically detectable monoclonal gammopathy but did not absolutely exclude a small or non-secretory B-cell or plasma-cell clone (16–18).

Accordingly, we describe this case as monotypic FGN without demonstrable monoclonal gammopathy rather than concluding that an underlying clonal process was definitively excluded. MGRS could not be established because no circulating monoclonal immunoglobulin or corresponding hematological clone was identified. Nevertheless, the presence of monotypic renal deposits warrants careful hematological evaluation and, when clinically appropriate, longitudinal surveillance.

This distinction is particularly important because confirmed DNAJB9-positive monotypic FGN is exceptionally rare and, unlike many other glomerular diseases with monotypic deposits, is not associated with detectable monoclonal gammopathy in most reported cases (16, 17).

### Relationship with rheumatoid arthritis

FGN occurring in the setting of rheumatoid arthritis has been described only rarely (13–15). In a multi-institutional cohort reported by Andeen et al., rheumatoid arthritis was present in 3 of 226 patients with FGN (approximately 1.3%) (13). Similarly, none of the 66 patients in the Mayo Clinic cohort had rheumatoid arthritis (15). To our knowledge, the previously published cases of FGN occurring in patients with rheumatoid arthritis did not demonstrate the combination of DNAJB9-positive FGN with confirmed IgG1-lambda restriction identified in the present case (19–21). The current case therefore expands the limited clinicopathological literature concerning FGN occurring in the setting of rheumatoid arthritis.

The mechanism underlying the observed association between FGN and rheumatoid arthritis remains uncertain. Chronic autoimmune and inflammatory disorders may be accompanied by persistent B-cell activation, altered immunoglobulin production, and dysregulation of immune responses, which could theoretically promote immunoglobulin-associated glomerular injury in susceptible individuals. Furthermore, the presence of IgG and DNAJB9 within the glomerular deposits of FGN has raised the possibility of an autoimmune component in its pathogenesis (11, 12). However, FGN is not currently regarded as a conventional immune-complex complication of rheumatoid arthritis, and no direct mechanistic or causal relationship between the two diseases has been established. Therefore, the coexistence of rheumatoid arthritis and monotypic FGN in our patient should be interpreted as a rare clinicopathological association rather than evidence that rheumatoid arthritis caused the glomerular disease.

### Differential diagnosis

The principal differential diagnoses include ITGN, amyloidosis, cryoglobulinemic glomerulonephritis, and PGNMID (4, 9). Their distinguishing ultrastructural, immunopathological, and clinical features are summarized in **Figure 3**. In this case, randomly oriented approximately 20-nm fibrils, diffuse DNAJB9 positivity, Congo red negativity, and absence of microtubular organization supported FGN. Concordant IgG1 and lambda restriction confirmed the monotypic phenotype (10, 11, 16, 17, 22).

**Figure 3:**
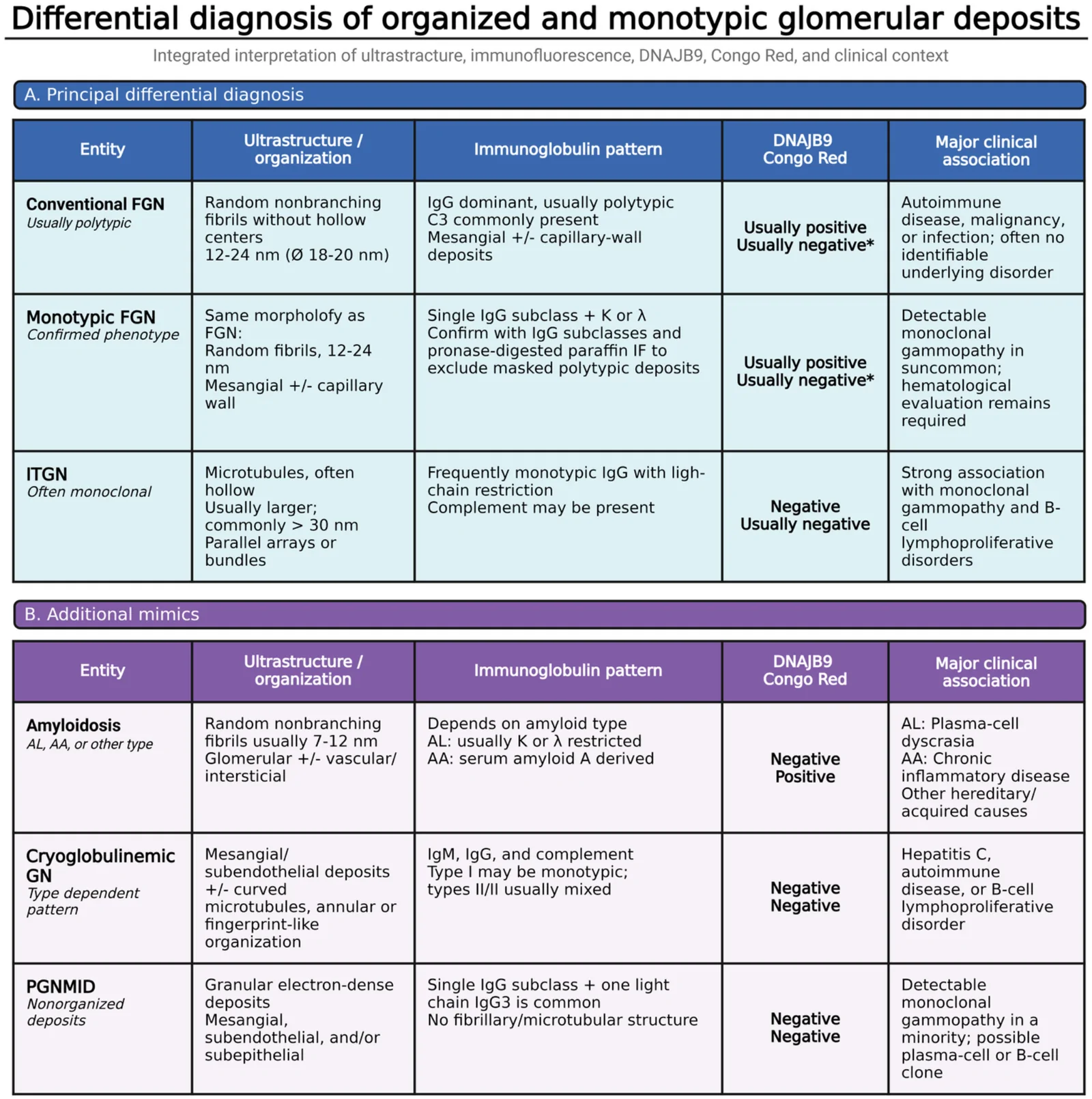
Differential diagnosis of organized and monotypic glomerular deposits *Rare Congo red-positive FGN has been reported. Monotypic staining does not by itself establish an underlying clonal disorder. FGN, fibrillary, glomerulonephritis; ITGN, immunotactoid GN; PGNMID, proliferative GN with monoclonal Ig deposits; IF, immunofluorescence.

### Role of DNAJB9

DNAJB9 is a member of the DnaJ heat-shock protein family and functions physiologically as an endoplasmic-reticulum co-chaperone involved in protein folding and the cellular response to misfolded proteins (11, 12). Proteomic studies identified DNAJB9 as a highly abundant component of FGN deposits, and immunoelectron microscopy subsequently localized the protein to the characteristic fibrils (12, 23). In renal biopsy tissue, IHC performed with an anti-DNAJB9 antibody typically demonstrates strong, smudgy staining of the mesangial and glomerular capillary-wall deposits. The term “anti-DNAJB9 antibody” in this context refers to the diagnostic antibody reagent used for tissue staining and should not be confused with circulating autoantibodies produced by the patient.

DNAJB9 immunohistochemistry has reported sensitivity of approximately 98% and specificity exceeding 99% for FGN (23). It is therefore a highly sensitive and specific diagnostic biomarker and is particularly useful in distinguishing FGN from amyloidosis, ITGN, and other glomerular diseases with organized deposits. However, DNAJB9 positivity should still be interpreted together with LM, IF, and EM findings. Rare DNAJB9-negative cases with otherwise compatible ultrastructural and immunopathological features have been reported, precluding its interpretation as an absolute diagnostic gold standard (24).

The biological role of DNAJB9 in FGN remains uncertain. Its colocalization with IgG1 and components of the classical complement pathway has led to the hypothesis that DNAJB9 may act as a putative autoantigen and participate in an IgG-mediated autoimmune response (12). Alternative explanations include binding of DNAJB9 to misfolded immunoglobulins or incorporation of the protein into fibrillary deposits through its normal chaperone function. These proposed mechanisms remain unproven. In the present case, DNAJB9 was demonstrated only as a tissue biomarker by IHC; circulating autoantibodies against DNAJB9 were not investigated.

### Clinical significance and treatment

No standardized treatment for FGN has been established. Limited retrospective and prospective evidence suggests that rituximab may stabilize kidney function or reduce proteinuria in some patients, although responses are inconsistent (11, 13, 14). In this patient, the reduction in proteinuria and increase in serum albumin were temporally associated with rituximab treatment. Treatment course and renal outcome is summarized in **Figure 4**. However, this uncontrolled observation cannot establish causality or demonstrate the general efficacy of rituximab in FGN.

**Figure 4:**
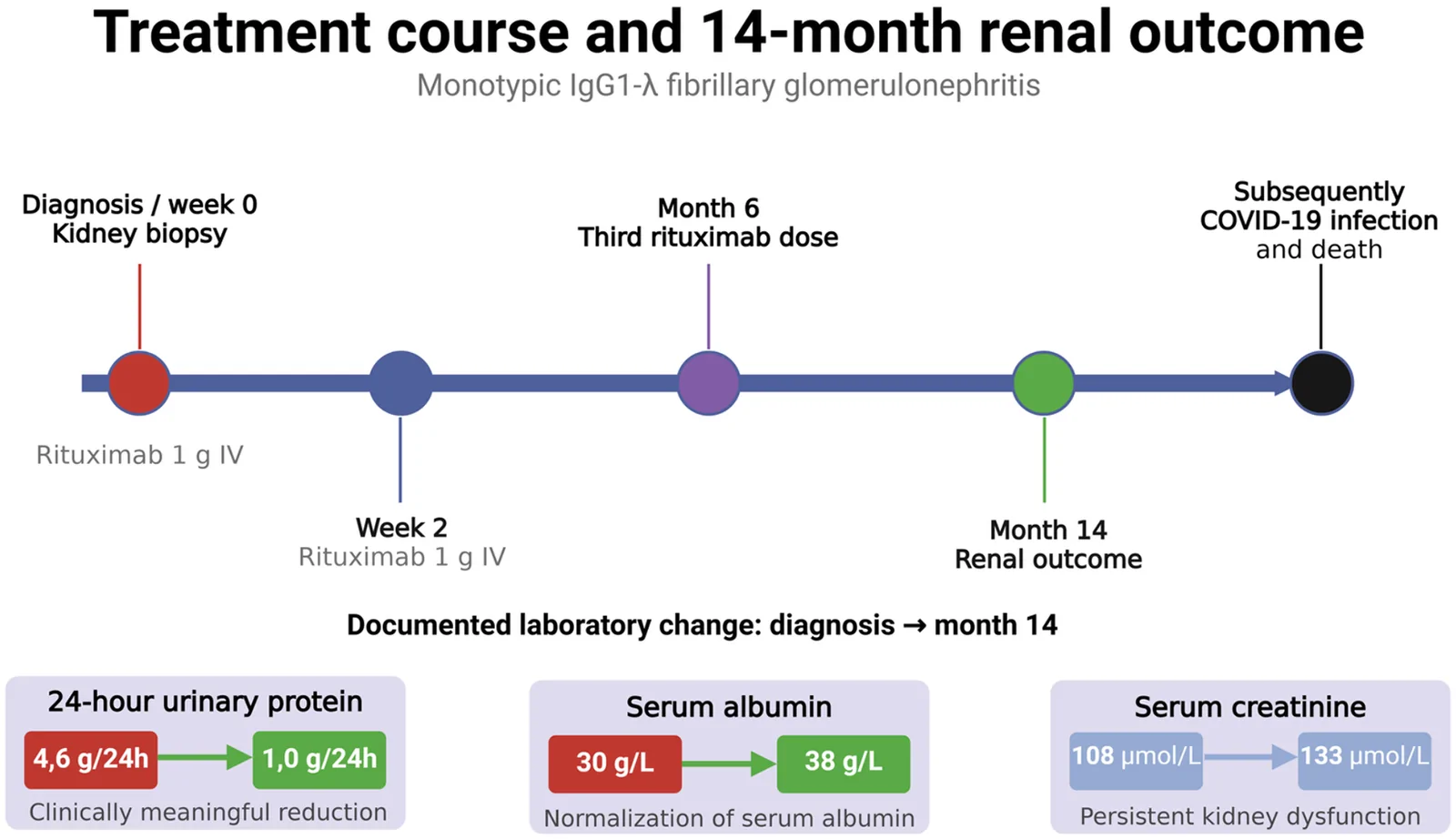
Rituximab treatment course and 14-month renal outcome. No intermediate laboratory measurements were available; values between diagnosis and month 14 were not interpolated. IV, intravenous; COVID-19, coronavirus disease 2019.

### Limitations

This report is limited by its single-patient design, incomplete intermediate laboratory measurements, absence of bone marrow examination and repeat kidney biopsy, and a follow-up restricted to 14 months by the patient’s subsequent death. Consequently, causality between rheumatoid arthritis and FGN, the presence of a small or non-secretory hematological clone, histopathological response, and the efficacy of rituximab cannot be definitively established.

## Conclusion

Monotypic FGN is exceptionally rare and requires integration of routine IF, IgG-subclass assessment, paraffin-embedded IF, DNAJB9 IHC, and EM. Light-chain restriction alone is insufficient to establish a truly monotypic process, and monotypic deposits warrant careful hematological evaluation without necessarily indicating an underlying clonal disorder. This case documents confirmed IgG1-lambda FGN occurring in the setting of rheumatoid arthritis without demonstrable monoclonal gammopathy.

## Data Availability

The original contributions presented in the study are included in the article/supplementary material. Further inquiries can be directed to the corresponding author.

## References

[1] Qadri AH Prajapati J Faheem I Bhattacharjee U Padmanaban HK Mulukala SKN . Biophysical characterization and insights into the oligomeric nature of CD2-associated protein. Int J Biochem Mol Biol. (2024) 15:20–33. doi: 10.62347/uvsh8436 38765876 PMC11101965

[2] Mulukala SKN Kambhampati V Qadri AH Pasupulati AK . Evolutionary conservation of intrinsically unstructured regions in slit-diaphragm proteins. PloS One. (2021) 16:e0254917. doi: 10.1371/journal.pone.0254917 34288970 PMC8294545

[3] Qadri AH Prajapati J Praghna D Sinha A Pasupulati AK . Structural and functional insights of the podocyte slit diaphragm complex. Tissue Barriers. (2026) 14:2575198. doi: 10.1080/21688370.2025.2575198 PMC13228966 41100809

[4] Jennette JC D'Agati VD Fogo AB Nickeleit V Stokes MB . Heptinstall’s Pathology of the Kidney Vol. 8. . Philadelphia, PA: Lippincott Williams & Wilkins (2023).

[5] Rosenmann E Eliakim M . Nephrotic syndrome associated with amyloid-like glomerular deposits. Nephron. (1977) 18:301–8. doi: 10.1159/000180846 865657

[6] Payan Schober F Jobson MA Poulton CJ Singh HK Nickeleit V Falk RJ . Clinical features and outcomes of a racially diverse population with fibrillary glomerulonephritis. Am J Nephrol. (2017) 45:248–56. doi: 10.1159/000455390 28161700 PMC5482172

[7] Rosenstock JL Markowitz GS . Fibrillary glomerulonephritis: An update. Kidney Int Rep. (2019) 4:917–22. doi: 10.1016/j.ekir.2019.04.013 31317113 PMC6611949

[8] Colvin RB Chang A Cornell LD . Diagnostic Pathology: Kidney Diseases. Philadelphia: Elsevier, 4th ed. (2023).

[9] Kudose S Canetta P Andeen NK Stokes MB Batal I Markowitz GS . Diagnostic approach to glomerulonephritis with fibrillar IgG deposits and light chain restriction. Kidney Int Rep. (2021) 6:936–45. doi: 10.1016/j.ekir.2021.01.001 33912743 PMC8071626

[10] Fogo A Qureshi N Horn RG . Morphologic and clinical features of fibrillary glomerulonephritis versus immunotactoid glomerulopathy. Am J Kidney Dis. (1993) 22:367–77. doi: 10.1016/s0272-6386(12)70138-5 8372831

[11] Klomjit N Alexander MP Zand L . Fibrillary glomerulonephritis and DNAJB9. Kidney360. (2020) 1:1002–13. doi: 10.34067/kid.0002532020 35369552 PMC8815599

[12] Andeen NK Yang HY Dai DF MacCoss MJ Smith KD . DNAJB9 is a putative autoantigen in fibrillary glomerulonephritis. J Am Soc Nephrol. (2018) 29:231–9. doi: 10.1681/ASN.2017050566 PMC5748922 29097624

[13] Andeen NK Troxell ML Riazy M Avasare RS Lapasia J Jefferson JA . Fibrillary glomerulonephritis: Clinicopathologic features and atypical cases from a multi-institutional cohort. Clin J Am Soc Nephrol. (2019) 14:1741–50. 10.2215/CJN.03870319 PMC6895488 31685544

[14] Erickson SB Zand L Nasr SH Alexander MP Leung N Drosou ME . Treatment of fibrillary glomerulonephritis with rituximab: a 12-month pilot study. Nephrol Dial Transplant. (2021) 36:104–10. 10.1093/ndt/gfaa065 32617582

[15] Nasr SH Valeri AM Cornell LD Fidler ME Sethi S Leung N . Fibrillary glomerulonephritis: a report of 66 cases from a single institution. Clin J Am Soc Nephrol. (2011) 6:775–84. doi: 10.2215/CJN.08300910 PMC3069369 21441134

[16] Said SM Leung N Alexander MP Cornell LD Fidler ME Grande JP . DNAJB9-positive monotypic fibrillary glomerulonephritis is not associated with monoclonal gammopathy in the vast majority of patients. Kidney Int. (2020) 98:498–504. doi: 10.1016/j.kint.2020.02.025 32622524

[17] Da Y Goh GH Lau T Chng WJ Soekojo CY . Fibrillary glomerulonephritis and monoclonal gammopathy: Potential diagnostic challenges. Front Oncol. (2022) 12:880923. doi: 10.3389/fonc.2022.880923 35692803 PMC9174543

[18] Leung N Bridoux F Batuman V Chaidos A Cockwell P D'Agati VD . The evaluation of monoclonal gammopathy of renal significance: a consensus report of the International Kidney and Monoclonal Gammopathy Research Group. Nat Rev Nephrol. (2019) 15:45–59. doi: 10.1038/s41581-018-0077-4 PMC7136169 30510265

[19] Yun YS . Fibrillary glomerulonephritis in rheumatoid arthritis. Nephrology. (2010) 15:266–7. doi: 10.1111/j.1440-1797.2009.01164.x 20470291

[20] Schreiber O . Fibrillary glomerulonephritis in a patient with rheumatoid arthritis: A case report and review of the literature. Clin Nephrol. (2024) 102:238–43. doi: 10.5414/cn111259 39037083

[21] Her MY . Fibrillary glomerulonephritis in a patient with rheumatoid arthritis. Clin Exp Rheumatol. (2008) 26:158. 18328168

[22] Rosenstock JL . Fibrillary and immunotactoid glomerulonephritis: Distinct entities with different clinical and pathologic features. Kidney Int. (2003) 63:1450–61. doi: 10.1046/j.1523-1755.2003.00853.x 12631361

[23] Nasr SH Vrana JA Dasari S Bridoux F Fidler ME Kaaki S . DNAJB9 is a specific immunohistochemical marker for fibrillary glomerulonephritis. Kidney Int Rep. (2018) 3:56–64. doi: 10.1016/j.ekir.2017.07.017 29340314 PMC5762944

[24] Shehzad P Khan SM Hodgins S . An atypical case of DNAJB9 negative fibrillary glomerulonephritis associated with COVID-19 infection. J Am Soc Nephrol. (2022) 33:338. doi: 10.1681/asn.20223311s1338a

